# Touchscreen-paradigm for mice reveals cross-species evidence for an antagonistic relationship of cognitive flexibility and stability

**DOI:** 10.3389/fnbeh.2014.00154

**Published:** 2014-05-05

**Authors:** S. Helene Richter, Anne S. Vogel, Kai Ueltzhöffer, Chiara Muzzillo, Miriam A. Vogt, Katja Lankisch, Diana J. N. Armbruster-Genç, Marco A. Riva, Christian J. Fiebach, Peter Gass, Barbara Vollmayr

**Affiliations:** ^1^Animal Models in Psychiatry, Department of Psychiatry and Psychotherapy, Central Institute of Mental Health, Medical Faculty Mannheim, Heidelberg UniversityMannheim, Germany; ^2^Bernstein Center for Computational NeuroscienceHeidelberg/Mannheim, Germany; ^3^Department of Psychology, Goethe UniversityFrankfurt am Main, Germany; ^4^Department of Pharmacological and Biomolecular Sciences, University of MilanMilan, Italy; ^5^Center for Individual Development and Adaptive EducationFrankfurt am Main, Germany

**Keywords:** cognitive flexibility, cognitive stability, touchscreen chambers, translation, mice, Dual State Theory, neurocomputational models, executive functioning

## Abstract

The abilities to either flexibly adjust behavior according to changing demands (cognitive flexibility) or to maintain it in the face of potential distractors (cognitive stability) are critical for adaptive behavior in many situations. Recently, a novel human paradigm has found individual differences of cognitive flexibility and stability to be related to common prefrontal networks. The aims of the present study were, first, to translate this paradigm from humans to mice and, second, to test conceptual predictions of a computational model of prefrontal working memory mechanisms, the *Dual State Theory*, which assumes an antagonistic relation between cognitive flexibility and stability. Mice were trained in a touchscreen-paradigm to discriminate visual cues. The task involved “ongoing” and cued “switch” trials. In addition distractor cues were interspersed to test the ability to resist distraction, and an ambiguous condition assessed the spontaneous switching between two possible responses without explicit cues. While response times did not differ substantially between conditions, error rates (ER) increased from the “ongoing” baseline condition to the most complex condition, where subjects were required to switch between two responses in the presence of a distracting cue. Importantly, subjects switching more often spontaneously were found to be more distractible by task irrelevant cues, but also more flexible in situations, where switching was required. These results support a dichotomy of cognitive flexibility and stability as predicted by the *Dual State Theory*. Furthermore, they replicate critical aspects of the human paradigm, which indicates the translational potential of the testing procedure and supports the use of touchscreen procedures in preclinical animal research.

## Introduction

The abilities to either flexibly adjust behavior according to changing environmental demands (cognitive flexibility) or to maintain it in the face of potential distractors (cognitive stability) form an important component of executive functioning (Diamond, 2013). Along with other higher-level cognitive skills, these abilities are crucial for our daily life when multiple behavioral options exist and demands are shifting (Banich, 2009). Deficits in these cognitive domains, however, are observed among patients suffering from psychiatric diseases (Dirnberger and Jahanshahi, 2013; Etkin et al., 2013; Snyder, 2013). Especially in schizophrenic patients, impairments in executive processing in general and in task switching or cognitive flexibility in particular have been reported (Wylie et al., 2010; Orellana and Slachevsky, 2013; Schirmbeck et al., 2013). Interestingly, impairments in the domains of higher-order cognitive functions are also found in non-affected family members of schizophrenic patients, indicating the presence of a genetic predisposition (Heydebrand, 2006; Snitz et al., 2006). Therefore, deficits in executive functioning may be considered core features of the disease that may even provide the basis on which other symptoms may occur (Barch, 2005; Beck and Rector, 2005).

From a neural perspective, these higher-level cognitive processes are supposed to involve various brain structures, most prominently the prefrontal cortex (PFC) and the mesocortical dopamine (DA) system (Goldman-Rakic et al., 2000; Winterer and Weinberger, 2004; Klanker et al., 2013). In line with these findings, both cognitive flexibility and cognitive stability have been related to PFC functioning (Curtis and D'Esposito, 2003; Floresco et al., 2009; Stelzel et al., 2010, 2013; Toepper et al., 2010; Kesner and Churchwell, 2011), but it is still not clear whether they depend upon separate or concordant neural networks. Therefore, a theoretical framework has been developed through a biophysically realistic computational model of the PFC-DA network that aims to identify mechanisms underlying cognitive flexibility and stability (Durstewitz and Seamans, 2008). According to this *Dual State Theory*, cognitive stability and cognitive flexibility are regulated by differential activity of the dopaminergic subsystems (D1-receptor-class vs. D2-receptor-class, respectively) in the PFC, possibly relying on differences in either receptor densities, baseline neurotransmitter levels, or the efficiency of neurotransmitter clearance (Durstewitz and Seamans, 2002, 2008; Bilder et al., 2004; Thurley et al., 2008). Specifically, simulation results led to the proposal of two distinct regimes, termed the D1-state and the D2-state, which either result in stable memory representations or flexible switching among representations, respectively. While in a D1-dominated state representations are characterized by a high energy barrier among different system states, resulting in representations that are robust to distraction (= cognitive stability), a D2-state results in low energy barriers between states, allowing for switching between representations (= cognitive flexibility). From a neurocomputational perspective, it can thus be derived that the degree of cognitive flexibility varies between persons due to, for example genetic differences in network properties or neurotransmitter levels, and that cognitive flexibility and stability are antagonistically related and controlled by a common neuronal network. Empirical data seem to support these predictions, in that working memory maintenance has been reliably associated with D1 mediated PFC activity (Goldman-Rakic et al., 2000) and some of our own work has shown that modulation of D2 signaling either genetically Stelzel et al.(2010) or pharmacologically Stelzel et al.(2013) affects the efficiency of cognitive flexibility.

**Figure 1:**
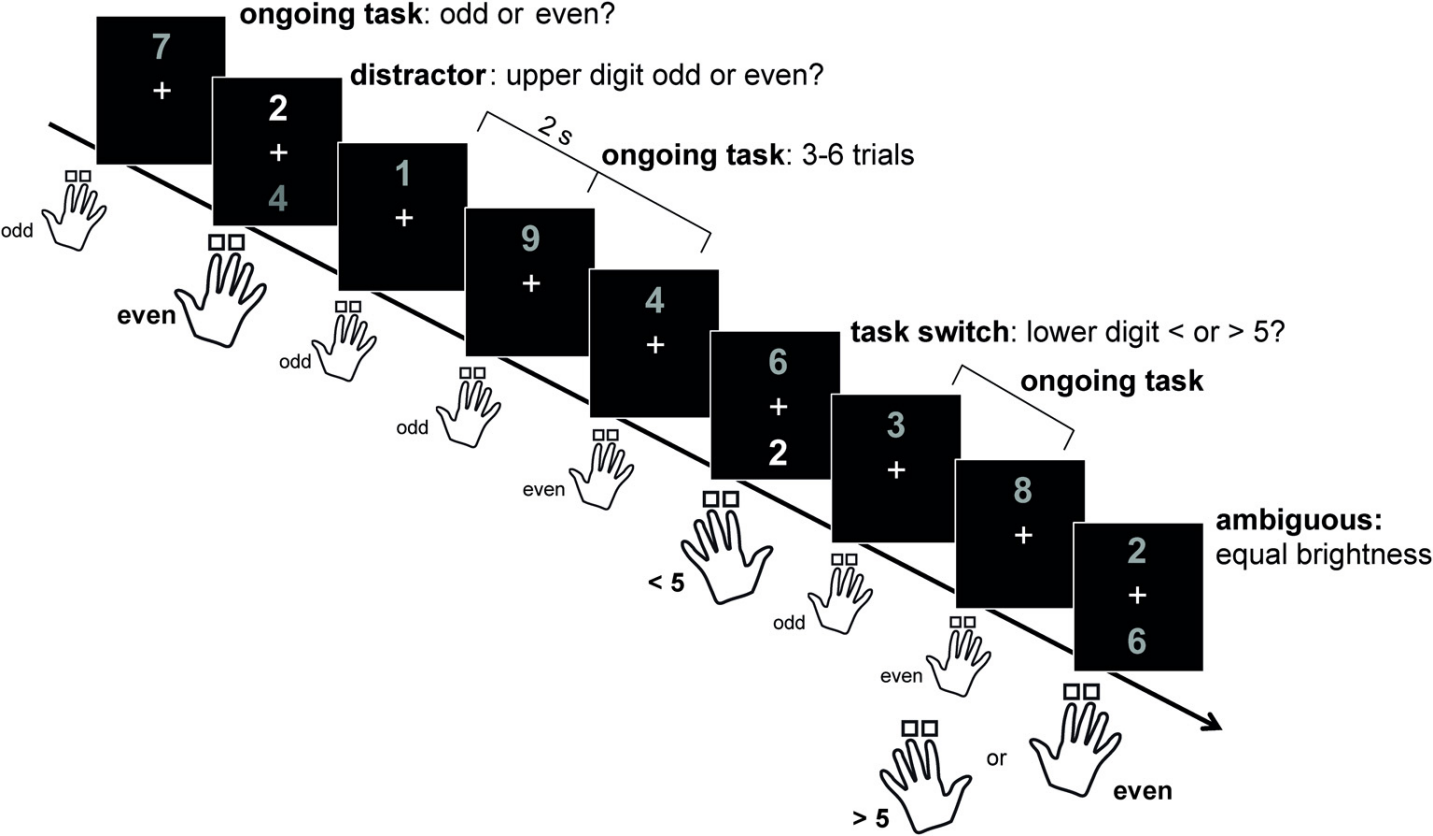
**The human STABFLEX test**. With permission from: Armbruster et al. (2012). Most of the trials required a response by button press to only one (upper) digit deciding wether it is odd or even. In 20% of the trials a second digit appeared below the fixation cross. Subjects had to ignore this digit if its color was darker than the upper one. If the lower digit was brighter however, subjects had to switch to the bottom digit and respond according to a different rule, i.e., >/<5 with the respective other hand (left and right hand counterbalanced between subjects). In ambiguous trials, brightness differences between stimuli were not detectable, and participants were free to switch or stay. This condition served to assess the individual spontaneous switching rate.

To systematically investigate the predictions made by the *Dual State Theory*, a novel task paradigm has recently been established in a healthy human population, which requires in the same task either cognitive flexibility or cognitive stability (Armbruster et al., 2012; Figure 1). Participants had to respond by button press to digits between 1 and 9. In most of the trials, only one digit was presented above a fixation cross and subjects had to decide whether this digit was odd or even (= rule 1). For the remaining 20% of trials, two digits were presented on the screen (above and below the fixation cross). If the upper digit was brighter than the lower digit, the participants had to continue using rule 1 and ignore the second distracting digit. By contrast, if the lower digit was brighter than the upper digit, subjects had to switch the rules and decide whether the lower digit was smaller or larger than 5 (= rule 2). Finally, in an ambiguous condition, the grayscale values of the two digits were indistinguishable, allowing for assessing the individual rate of spontaneous switching in the absence of explicit external cues, as a measure of individual differences in cognitive flexibility (Armbruster et al., 2012). Participants were found to differ substantially in the individual spontaneous switching rate, and more flexible persons were more efficient in task switching but also more distractible during distractor inhibition. These results support the dimensional model of cognitive flexibility and stability described by the *Dual State Theory* (Durstewitz and Seamans, 2008).

However, not all predictions of computational models can be adequately addressed in human subjects. Translational approaches are necessary to understand cellular and molecular mechanisms and identify new treatment options (Van Der Worp and Sandercock, 2012; Homberg, 2013). A high failure rate of preclinically identified compounds in the clinical trials (Kola and Landis, 2004) underscores the importance of reliability and validity of animal models as a premise to draw meaningful translational conclusions from preclinical findings. Some biomedical research efforts therefore now aim at translating clinical findings back to measures in animal disease models (e.g., Garner et al., 2006).

Therefore, the aim of the present study was to introduce a mouse paradigm to assess cognitive flexibility vs. cognitive stability in close analogy to the human paradigm using cues and outcome measures of the same qualities (Armbruster et al., 2012). In contrast to already established set-shifting procedures in mice (Garner et al., 2006; Endo et al., 2011; Bissonette and Powell, 2012; Scheggia et al., 2014), the strength of the present approach is twofold: First, it has a high translational value as it is derived from a behavioral paradigm for humans, and second, it is conceptually based on a biophysically plausible neurocomputational theory that has the potential of linking animal and human behavior across species.

In analogy to the human paradigm, we assessed within one paradigm the individual's task performance in the presence of irrelevant distractor cues and the flexibility required when responding to switching cues, as well as the individual's disposition to spontaneously switch in response to ambiguous stimuli. According to the dimensional model of Durstewitz and Seamans (2008) and in line with the human data, we expected to find opposing trends in behavior among individuals: More flexible subjects with a greater tendency toward switching in an ambiguous situation were thus expected to switch faster and more accurately when explicitly cued, while at the same time being more prone to distraction. By contrast, more stable subjects characterized by less spontaneous switching behavior were expected to resist distraction more efficiently, while at the same time making more errors when cognitive flexibility (i.e., switching) is explicitly required.

In sum, the present study aims at translating a human stability-flexibility paradigm to mice. Via comparison of behavioral results from mice to those previously derived from humans, we both validate the animal paradigm and at the same time provide supportive evidence for the *Dual State Theory* from a different species, i.e., mice.

## Animals, materials, and methods

### Animals

The subjects were 24 male C57BL/6N mice (Charles River, Sulzfeld, Germany), approximately 13 weeks old at the onset of the experimental procedure. Mice were single-housed in conventional macrolon cages (Type II, 26 × 20 × 14 cm) with sawdust (Rehofix MK-2000; Rettenmaier & Söhne, Rosenberg, Germany), nesting material, and tap water *ad libitum*. Upon arrival all animals were earmarked with individual patterns to allow precise identification of the individual mouse. Once per week, the cages were cleaned, water bottles replaced, and new tissue paper provided. The colony room was maintained at a temperature of 23 ± 2°C, a relative humidity of 50 ± 5% and a reversed 12 h light-dark schedule with the lights off at 7 am.

Prior to testing, *ad libitum* feeding weights were obtained, and mice were food-restricted to 85–90% of their initially measured individual bodyweight. To maintain the animals in a healthy state and to adjust the daily amount of food individually, the weight and health status of each mouse was checked on a daily basis. Food restriction, however, did not cause any observable changes in the animals' behavior.

All experiments complied with the regulations covering animal experimentation within the EU (European Communities Council Directive 2010/63/EU) and were approved by German animal welfare authorities (Regierungspräsidium Karlsruhe). Moreover, all efforts were made to minimize the number of animals used and the severity of procedures applied in the study.

### Test

#### Apparatus

**Figure 2:**
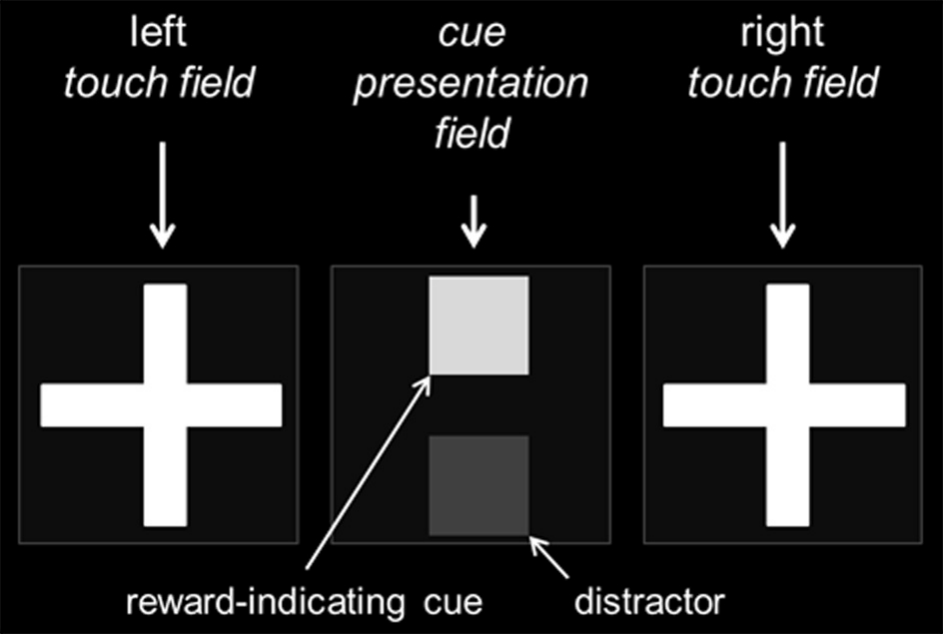
**Organization of the cue-presenting and touch-sensitive fields**. The touchscreen was covered by a black Perspex mask with three equal response windows. While the central field served as the cue presentation field, the external fields were used as touch fields during training and testing. For the performance of the “cue-position task,” a reward-indicating cue was presented either in the top or bottom position of the central cue presentation field and the mouse had to respond by touching either on the left or right cross in the touch fields (position-to-side assignment balanced across subjects). Dependent on the test condition a distractor of a different gray value could be displayed simultaneously to the reward-indicating cue in the central cue presentation field.

All animals were tested in Campden Instruments Ltd. (Loughborough, Leics., UK) mouse touchscreen chambers (Model 80614-20). The chambers were equipped with a 3-W house light, a tone generator and several light beams detecting the movement of the mouse. The trapezoid shaped inner chamber (h 19 cm, w 24 respectively 6 cm, d 17 cm) consisted of black Perspex walls and a metal grid floor. At one end, the boxes were equipped with screens surrounded by infra-red detectors to sense touches. As a consequence, the mice were not required to get in direct contact with the screen, but needed to approach the stimuli closely by nose-poke responses. The screens were partly covered by a Perspex mask in order to block access to the display except through three equal response windows that measured 7 × 7 cm each (Figure 2). Each window was separated by black Perspex dividers to prevent accidental approaches to the adjacent response window. During the training and testing procedure the outer fields were used to detect touches (left and right *touch fields*), whereas the center field was used to present cues (*cue presentation field*) (Figure 2). A food well (2 × 2 × 2 cm^3^) attached to an externally placed feeder (liquid suspensor) was located centrally at the rear of the chamber. A panel light illuminated the food tray and head entries were detected via a light beam detector. Here, mice were also trained to initiate a trial by breaking the light beam.

#### Procedure

Using touchscreen chambers, we translated the above described human paradigm (Armbruster et al., 2012) as analogously as possible to the mouse condition. In brief, the mouse-translation of the human paradigm involved side rather than task switches, with repetition trials (“ongoing”) occurring with equal likelihood on both sides. A central field was used for the illustration of a reward-indicating cue that could be presented in three different gray intensities in two different positions. According to the position of the cue, the mouse had to touch either on the left or the right *touch field* to get a reward. In some trials, two cues of different gray intensity were presented simultaneously. In these trials, the animal had to solve the task according to the brighter cue, thus ignoring the second distracting cue. In an ambiguous condition two cues of the same gray intensity were presented and the mouse could choose freely between the responses. Thus, taking species-specific differences into account, we modified the test with respect to five key points:

1. Humans can be instructed and tested in a single session on 1 day, while rodents need to learn various preparatory tasks in an extensive training, before being capable of managing the final test. Thus, the time needed to complete the testing is far greater in the murine than in the human test version.
2. To guarantee a high degree of motivation throughout testing, a rodent test version inevitably relies on a reward-based structure. Thus, rewarding correct behavior and punishing incorrect answers are fundamental strategies in the mouse test version. Moreover, animals need to be food restricted prior to and during the testing to set up the conditions for testing.
3. In the human paradigm, subjects are asked to respond to a visual task by pushing a button with one of four possible fingers (i.e., two fingers per task), while mice have to make a choice by responding directly to the touch-sensitive screen via nose-pokes. Because of the different locations of the *touch fields* this behavioral response contains a larger motoric component that may influence the output measures (e.g., response times).
4. While the human task required the subject to switch between two different rules depending on the position of a cue (cue above the fixation cross > rule 1, cue below the fixation cross > rule 2), the mouse task relied on a simpler discriminative task (Is the cue in position one or two? > touch left or right). Because only the change between two different rules within one test is conventionally considered to be a “task switch” (Brigman et al., 2005; Garner et al., 2006), the mouse test version did not include a “task switch” by definition.
  However, as the aim of the present work was to investigate the antagonistic nature of cognitive flexibility vs. cognitive stability according to the *Dual State Theory* (and not task switching *per se*) a task switch was not necessarily required. As cue-guided switching of response position within one task may in itself be a demanding challenge on the cognitive systems of a mouse, we replaced the human task switching aspect of the paradigm by the requirement of flexible or stable responses to a discriminative task. Therefore, the procedure was simplified to increase efficacy and practicability in a mouse model without compromising specificity.
5. Since the human paradigm included fMRI imaging in the experimental setup, it was necessary to determine a baseline state from which differences due to behavioral responses could be monitored. The baseline task (= responding to a digit shown in the “upper” position) was presented in 80% of all cases in order to maintain the brain in a steady state. In 20% of the cases a second digit was presented simultaneously in the “bottom” position to allow for assessing brain activation changes under theoretically interesting conditions, i.e., stressing either cognitive flexibility or stability or, in the ambiguous condition, providing no clear cues. An exact translation of this paradigm to the mouse paradigm, however, would have led to a repeated presentation of one cue in the same position, requiring the mouse to respond in always the same way. Thus, to avoid the risk of activating a side bias, animals were trained to both cue positions equally.

**Figure 3:**
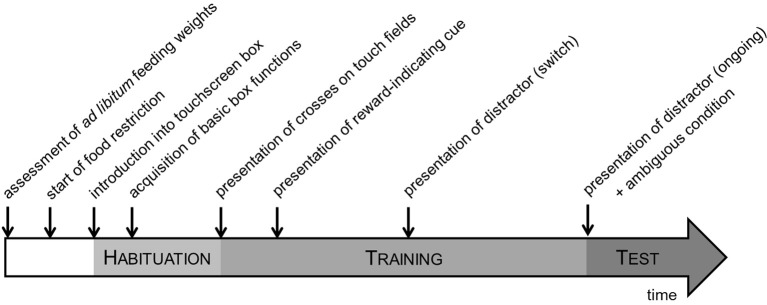
**Experimental time schedule**. The testing procedure was divided into three main phases: habituation, training and testing phase. During the habituation and training phases, mice were acquainted with the basic box functions and learned to respond to the discriminative task before being tested in the STABFLEX test.

The mice were housed under stable conditions for 4 weeks before the onset of testing. The testing procedure was divided into three main phases: habituation, training and testing phase (Figure 3). The daily testing order followed a fixed schedule to guarantee a consistent level of motivation due to feeding times. At the beginning of each test session, mice were transported to the test room in their home cages and allowed to acclimatize to the room before testing commenced. The testing was done during the dark phase of the cycle, 2 h after the light change. Food was supplied individually following the testing procedure. Inner chambers were cleaned after the testing of each animal with water. One week ahead the first introduction into the boxes, the animals received sweet condensed milk (SCM) in their home cages in order to avoid later refusal of the reward provided in the touchscreen boxes.

***Habituation***. During the habituation phase the mice learned basic procedures inside the box, such as touching on the screen, getting a reward, or initiating the next trial (Figure 3). Because the procedures have been described in detail elsewhere (see Talpos et al., 2009), we limit the following description to a brief overview: First, subjects were habituated to the boxes once a day with increasing duration (10–40 min) on three consecutive days. During the second session, a liquid reward (7 μl SCM “Milchmädchen,” diluted 1:4 in tap water) was placed in the food well that was freely accessible to the subjects. In the third session, mice learned to associate the supply of the reward with the illumination of the food well-light and the presentation of a tone. Following this, they became acquainted with symbols presented on the *touch fields* and learned to nose-poke them to receive a reward. In the next habituation step the animals learned to initiate each trial by breaking a light beam within the feeder well to produce the stimulus onset. A correct nose-poke to the response screen was followed by the disappearance of the stimuli, presentation of a tone and delivery of the reward. Initiation of the next trial could then occur after a 5 s inter trial interval (ITI). Finally, punishment of incorrect answers was introduced in form of a 5 s timeout with house light illumination before the start of the ITI.

***Training***. In the training the animals were required to learn a rule of the type “if cue on *cue presentation field* in position A then go left, if cue on *cue presentation field* position B then go right” (Figure 3). The position-interpretation was consequently counterbalanced across animals. The training was subdivided into four phases which build on each other introducing single items of the final task needed in the testing. The position-sequence was determined pseudo-randomly based on phase-dependent variations of probability of occurrence.

Phase 1 was named “two crosses, no cue”: A nose-poke to crosses presented on *touch fields* was rewarded with a tone, feeder light and a liquid reward. Touches on the *cue presentation field* did not lead to any reaction of the system. The criterion to progress into the next phase was performance of 30 trials in 30 min. In the second phase “Cue-Position Task” a cue on the *cue presentation field* in either top or bottom position indicated which side was counted “correct” and rewarded when touched. The cue was a gray square (2.5 cm) presented in one of three different possible light intensities. Top and bottom position were separated by a gap of 2.0 cm from the boundaries of the squares. Only touches on the *touch field* lead to supply of a reward. Each session included 40 trials or was terminated after 60 min. The criterion to progress into the next phase was performing 40 trials per day on two consecutive days. In 50% of all trials the position of the cue did not change between two consecutive trials (“ongoing” condition), while it changed between the trials in the other 50% (“switch” condition). Touches on the non-reward-indicating *touch field* and the *cue presentation field* did not result in any reaction of the system. During the “Cue-Position Task with correction”-phase touches on the non-reward-indicating *touch field* were counted “incorrect” and lead to a 5 s timeout with house light illumination and initiation of a correction trial, which was counted separately and repeated until the animal responded correctly. Each session consisted of 40 trials (not including correction trials) with equal occurrence probabilities for “ongoing” and “switch” conditions. The session was either finished after the completion of 40 trials or a training time of 60 min. The criterion to progress into the next phase was performance of 40 trials with at least 80% correct responses on two consecutive days.

**Figure 4:**
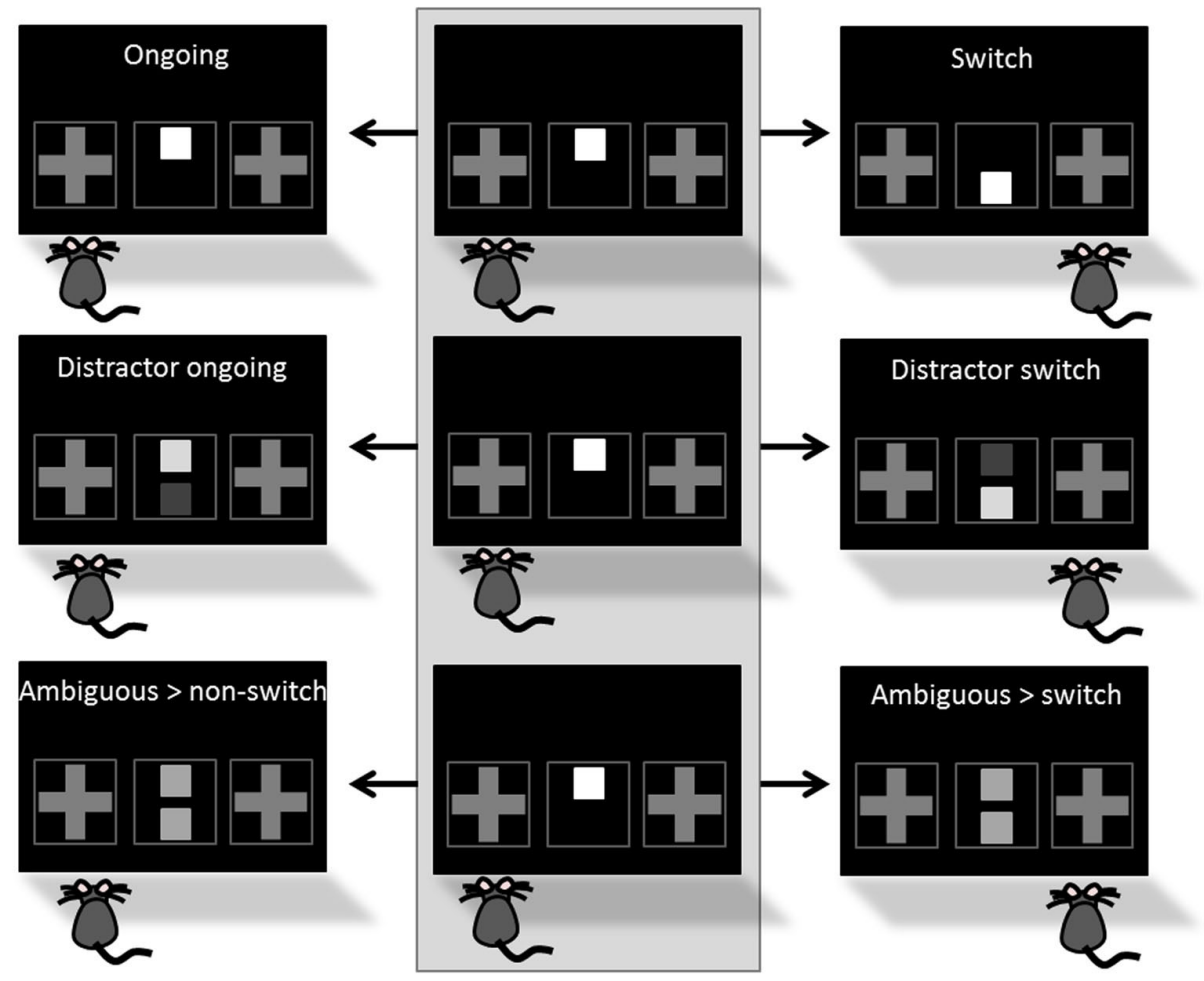
**Task conditions**. The STABFLEX test combines five different task conditions, including ongoing, switch, distractor ongoing, distractor switch, and ambiguous conditions. Different conditions are defined by the number of cues presented in the cue presentation field (one or two), their position (top or bottom), their relative intensity (different gray values), and the foregoing trial (middle column colored in light gray). **Top row**: While an ongoing trial is characterized by the presentation of only one cue in the same position in two consecutive trials (left), a switch condition describes a change in the position of the cue between the trials (here: cue at the top > cue at the bottom, right). **Middle row**: The distractor conditions, i.e., distractor ongoing (left) and distractor switch (right), are based on the same principle, but are complemented by a second cue of different intensity that is task irrelevant and thus a distractor that renders the task more difficult. For answering correctly, the animal needs to solve the task according to the brighter cue. **Bottom row**: Finally, animals are presented with ambiguous situations that are characterized by the presentation of two cues of the same intensity. The classification of these trials as ambiguous/non-switch (left) vs. ambiguous/switch (right) depends on the behavior of the animal and this condition does not favor one of the two response alternatives. Conditions are presented as a series of two consecutive trials, starting with the foregoing trial in the middle column of the illustration and choices are depicted by the position of the mouse in front of the screen.

In the last training phase “Cue-Position Task with distractor and correction,” the “distractor switch” condition was introduced. As in the “switch” condition the reward-indicating cue occurred in different positions on two consecutive trials, but was additionally accompanied by the presentation of a distracting cue in the second trial (Figure 4). The distracting cue (= distractor) occurred in form of a second gray square on the *cue presentation field* and was presented simultaneously to the reward-indicating cue. Similar to the reward-indicating cue the distractor was presented in three different gray intensities, all darker than the reward-indicating cue and for answering correctly, the mouse needed to solve the task according to the brighter cue. Combination of brightness intensities of the two cues consistently equaled the same mean brightness. “Ongoing,” “switch,” and “distractor switch” conditions were presented with occurrence probabilities of 50, 25, and 25%, respectively. Incorrect touches (touches according to the distractor) led to a 5 s timeout with house light illumination, followed by correction trials. Initially, the animals had to perform 40 trials a day which was subsequently raised to 50 trials. In both phases the learning criterion was set to 80% correct responses on two consecutive days.

***Testing***. Once a mouse had successfully reached the final learning criterion, it was tested in the translational stability/flexibility (STABFLEX) test on 14 consecutive days. A testing day consisted of 50 trials per day and mouse, yielding a total amount of 700 trials per animal. A single session ended after the performance of 50 trials or 60 min elapsed time. In contrast to the training phase, testing did not contain any correction trials, while incorrect responses were still punished with house light illumination and 5 s timeout. For all testing days, we used pseudo-randomized sequences with no more than three consecutive “ongoing” trials. Between the “ongoing” conditions one out of four possible other conditions were presented. All conditions, namely “switch,” “distractor switch,” “distractor ongoing,” and “ambiguous” (Figure 4) had the same probability to appear (probability of condition appearance: 66% ongoing, 8.5% each switch, distractor switch, distractor ongoing and ambiguous). While “switch” and “distractor switch” conditions were introduced in the training, the “distractor ongoing” and “ambiguous” conditions were completely new to the mice in the testing procedure (Figure 3).

In the “distractor ongoing” condition a distractor of another gray intensity was presented simultaneously to the brighter reward-indicating cue on the *cue presentation field* (Figure 4; **middle row**). As in the “distractor switch” condition, the subject was required to follow the lighter cue and answer according to the “ongoing” condition. In the “ambiguous” condition two squares with identical gray intensity were presented on the *cue presentation field* (Figure 4; lower row). In this condition, the task did thus not unambiguously indicate a rewarded and a non-rewarded side, so that the animal could freely choose between the two possible responses. In any case, however, the choice was followed by a reward.

#### Behavioral measures

While in the training phase the number of trials required to attain criterion performance in the different training phases was of major interest for the analysis, performance in the STABFLEX test was assessed using the following behavioral measures: (i) number of errors [error rates (ER)], (ii) average choice latencies or response times for correct responses (RT), which were measured as the time from the onset of the cue presentation until the mouse made a nose-poke response; and (iii) response time costs, which refer to the difference between the RTs of any condition and the baseline condition (“ongoing”). In addition, two further behavioral measures were calculated, the “distractor resistance” and the “individual spontaneous switching score,” to analyze the data as analogously as possible to the human condition.

First, a so-called “distractor resistance” was calculated with the aim of describing an animal's ability to maintain a behavior in the face of potential distractors. As more stable subjects are expected to be more resistant to distraction (Durstewitz and Seamans, 2008; Armbruster et al., 2012), the measure was based on correct and incorrect choices made in the “distractor ongoing condition.” More precisely, distractor resistance was calculated as a choice score (correct choices—incorrect choices) corrected for the overall individual performance in the “ongoing” condition. By including the correction for the overall success rate under baseline conditions (“ongoing”) in the formula, inter-individual differences in learning abilities were taken into account. On a scale from −1 (very distractible) to +1 (resistant to distraction) this score describes an individual's ability to resist distraction.

$$\begin{array}{l} distractor\;resistance\\ =\frac{N_{distractor}\;\left(correct\right)-\;N_{distractor}\;\left(incorrect\right)}{N_{distractor}\;\left(correct\;+\;incorrect\;choices\right)}\;÷success\;rate_{ongoing} \end{array}$$

According to the human test version (Armbruster et al., 2012), the probability of spontaneously switching under ambiguous conditions without external cues was also assessed. This so-called “individual spontaneous switching score” (ISSS) was calculated on the basis of switches and non-switches in the “ambiguous” condition:

$$ISSS=\;\frac{N_{ambiguous}\left(switches\right)-\;N_{ambiguous}(nonswitches)}{N_{ambiguous}\;(switches+\;nonswitches)}$$

Only responses which followed a correct response in the preceding “ongoing” condition were included in this analysis. Similar to the “individual spontaneous switching rate” in the human paradigm, this measure reflects the tendency of an animal to either continue responding with the same behavioral response (stability) or to switch between the responses and exhibit more flexible characteristics. On a scale from −1 (very stable) to +1 (very flexible) this score thus describes an individual's tendency to behave in a stable or flexible way.

### Data analysis

Behavioral log files of the conducted experiments contained full information on the course of the experiments. Each trial was represented by a matrix row containing the experimental condition, the response given and the response time. This data was reduced by averaging the latencies over specific condition × decision combinations and by counting the numbers of specific responses as a function of experimental condition and in case of the ISSS also as a function of the condition and response in the preceding trial (see Behavioral Measures). These calculations were performed using custom MATLAB routines.

Latency measures and ER of 22 mice were then entered into the statistical analysis presented below. Latencies were determined by averaging all response times of one condition across all correct trials of this mouse, while errors were summed up per condition and related to the amount of trials per condition to determine individual ER. For inferential statistics, repeated measures ANOVAs were applied to investigate the effects of “condition.” on response times and ER. When necessary, *post-hoc* tests were performed using a Bonferroni correction. To test the predictions made by the *Dual State Theory*, correlation analyses were conducted using Pearson's product-moment correlation coefficient (*r*) and Spearman's rank correlation coefficient (*r*_*s*_). All statistical tests were conducted using the software package SPSS (version 19.0 for Windows), and differences were considered to be significant at *p* ≤ 0.05.

## Results

### Habituation and training

Of the 24 mice that were used for the establishment of the STABFLEX test, 22 individuals were entered into the following analyses. The exclusion of two animals was based on either bad or inconsistent performance in the *cue position task* or in the testing phase. Thus, one subject was excluded from the analysis, because its overall success rate did not exceed chance level in the *cue-position task*, although it was trained for 86 days. The second animal succeeded to go through the various training phases, but stopped behaving in an active way after a few days in the testing phase. Because the subject did not show any behavioral abnormalities in the home cage and we could not observe any signs of disease, we considered this behavior to have a motivational cause and excluded the animal due to a lack of data.

**Figure 5:**
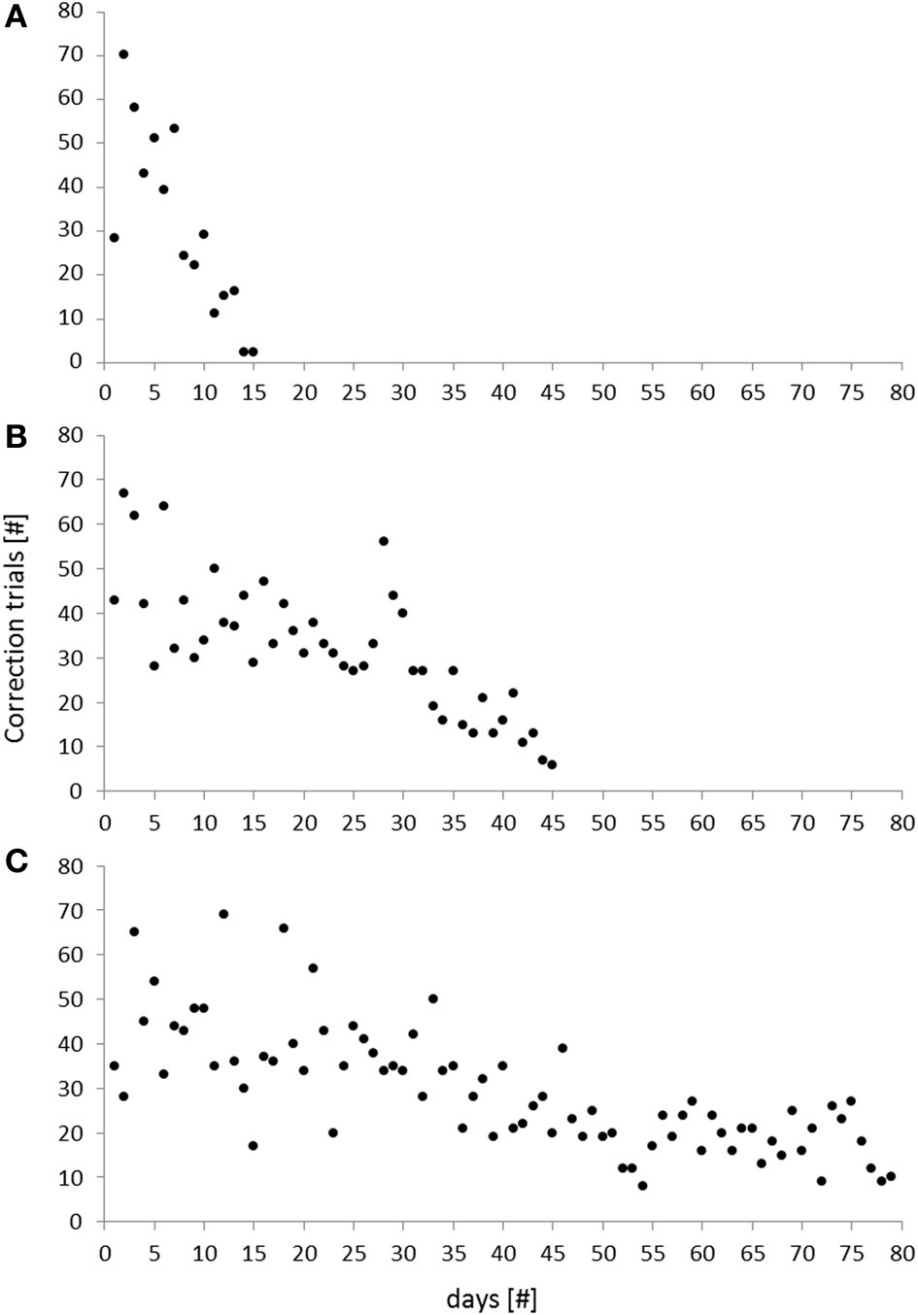
**Number of correction trials during the cue-position task with correction of three individual mice**. The total number of correction trials turned out to be a good measure for an individual's learning progress in this phase. Different types of learning curves were observed: While some subjects were characterized by a rapid decrease of correction trials, reflecting a steep learning progress **(A)**, others were characterized by a more gradual learning improvement over time **(B,C)**.

During the initial phase of the task (habituation) animals were habituated to the touchscreen-box and required to learn how to initiate trials and to touch on the screen for getting a reward. On average, mice needed 15 days to go through these basic steps with only two animals that needed more than 20 days to reach the criterion (Table 1). The subsequent training phase consisted of five different sub-phases that were completed after 63 ± 4 days. Notably, the overall training duration ranged from 29 to 90 days, reflecting considerable inter-individual variability (Table 1). Differences in training duration, however, were mainly due to performance differences in the *cue-position task with correction*. While one mouse reached the criterion already after 15 days, another one required 79 days to go through this critical training phase (Table 1). In this context, the number of correction trials per day turned out to be a good measure for the assessment of an individual's learning progress throughout this phase (Figure 5). While some individuals were characterized by a rapid decrease of correction trials (Figure 5A), the performance of other individuals followed a more gradual improvement over several days (Figure 5C). Thus, although some animals required more than 2 months learning the *cue-position task with correction*, their performance was characterized by a continuously decreasing number of correction trials.

**Table 1:** **Duration of habituation and training phases in days**.

	**Habituation**	**Training (cue-position task with correction)**
Mean	15	63 (47)
s.e.m	1	4 (4)
Min.	12	29 (15)
Max.	25	90 (79)

*Subjects had to reach specific learning criteria to enter the next phase. Accordingly, habituation and training durations differed widely between the individuals, n = 22*.

Furthermore, we were interested in the question whether a “good performer” in the training phase remains a “good performer” in the test phase. We therefore correlated the number of days needed to go through the *cue-position task with correction* in the training with the overall success rate in the testing phase. Indeed, we found a significant negative correlation between the duration of this training phase and the later success rate: The faster an animal reached the learning criterion in the *cue-position task with correction*, the better it was in the testing phase (*r* = −0.493, *p* = 0.020; *r*_*s*_ = −0.459, *p* = 0.031).

### Testing cognitive flexibility vs. cognitive stability

**Figure 6:**
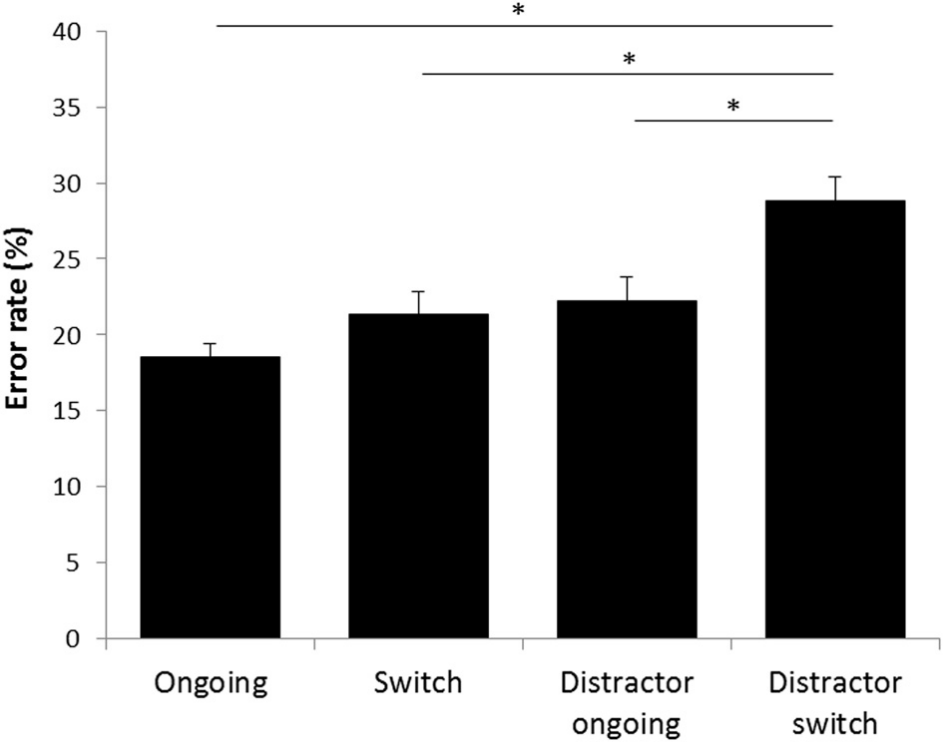
**Error rates in the STABFLEX test**. The error rates were calculated on the basis of the number of incorrect touches divided by the total number of trials per condition. Conditions were compared using a repeated measures ANOVA with “condition” as within-subjects factor and subsequent Bonferroni-corrected *post-hoc* analyses. Data are presented separately for the four experimental conditions as means ± standard error of the mean. ^*^p ≤ 0.05.

Testing was successfully done with 50 trials per day on 14 successive days in all mice, yielding a total amount of 700 trials per animal. Although animals were not required to reach a specific learning criterion during the testing phase, the overall success rates were similar to those of the final training phase, indicating robust performances over time. With a mean of 79.3% the overall success rate (= correct choices/700) ranged from 67.5 to 89.1% (Table 2). Except of one animal that did not reach the 70% mark, mice solved the task correctly in at least 75% of all trials, demonstrating constantly good performance abilities also during the testing phase. In addition to the overall success rate, behavioral performance in the testing phase was separately analyzed for each condition (“ongoing,” “switch,” “distractor ongoing,” “distractor switch,” “ambiguous”) using both response times and ER. Concerning the response times, only minimal differences were observed between the five testing conditions, ranging from a mean latency of 1.9 s in the “distractor ongoing” condition to 2.1 s in the “ambiguous” condition (Table 3). Accordingly, the observed differences between response times of the specific event conditions and the “ongoing” condition did not exceed ± 130 ms. Notably, we observed negative response time costs in the distractor conditions, indicating that on average it took the mice slightly longer to respond to the “ongoing” than to the “distractor ongoing” and the “distractor switch” conditions (Table 3). By contrast, ER clearly differed between the conditions in the expected way, with only 18.6% in the “ongoing” condition and 28.8% in the “distractor switch” condition, probably reflecting an increasing degree of difficulty (Figure 6).

**Table 2:** **Summary of main behavioral measures**.

	**Success rate (%)**	**Individual spontaneous switching score**	**Distractor resistance**
Mean	79.3	−0.057	0.698
s.e.m	1.0	0.041	0.037
Min.	67.5	−0.302	0.293
Max.	89.1	0.349	0.944

*On the basis of correct and incorrect choices in five different test conditions, three main behavioral parameters were calculated: overall success rate (correct choices independent of the specific task condition), individual spontaneous switching score, and distractor resistance, n = 22*.

**Table 3:** **Descriptive statistics for response times (RT), and response time costs**.

	**Response times (ms)**	**Costs (ms)**
	**Mean ± s.e.m**.	**Mean ± s.e.m**.
Ongoing	2055±116	
Switch	2057±121	2±58
Distractor ongoing	1926±97	−130±61
Distractor switch	2022±118	−33±46
Ambiguous	2104±118	49±50

*While the absolute response times capture mean latency values per condition, the costs are corrected by the time needed to finish an ongoing trial (e.g., RT switch − RT ongoing = Switching costs). Data are separately presented for each test condition and presented as means ± standard error of the mean, n = 22*.

Subsequent inferential statistics, using repeated measures ANOVAs, revealed significant effects of the condition on both response times [*F*_(4, 84)_ = 2.775, *p* = 0.032] and ER [*F*_(3, 63)_ = 12.267, *p* < 0.001]. Further Bonferroni corrected *post-hoc*-analyses of the response times, however, showed that differences were only approaching significance and restricted to the comparison of the “ambiguous” condition with the “distractor ongoing” condition (*p* = 0.06), while all other conditions did not differ significantly. With respect to the ER, a significant difference was found between the “ongoing” and “distractor switch” condition (*p* < 0.001, Figure 6), while there were no statistically significant differences between “ongoing,” and “switch” and “ongoing,” and “distractor ongoing” conditions (*p* > 0.1). Furthermore, ER of the “distractor switch” condition were higher than those of the “switch” condition (*p* < 0.001) and those of the “distractor ongoing” condition (*p* = 0.034, Figure 6). A difference between ER of the “switch” and the “distractor ongoing” conditions was not observed (*p* > 0.1).

To test some behavioral predictions derived from the *Dual State Theory*, two further behavioral measures were investigated: The ISSS and the distractor resistance (see Animals, Materials, and Methods). While the ISSS aims to capture the individual level of flexibility/stability in the “ambiguous” condition on a scale from −1 (extremely stable) to +1 (extremely flexible), the distractor resistance was calculated on the basis of correct and incorrect choices in the “distractor ongoing” condition and as such reflects an individual's ability to solve a task correctly in face of distracting stimuli (the higher the value, the more stable the individual, see Animals, Materials, and Methods). Both measures were found to vary substantially between individuals, indicating different degrees of cognitive flexibility and stability in our sample (Table 2). Similar to the previous human study, the ISSS thus allowed for identifying more stable (<0) and more flexible (>0) subjects. Furthermore, we observed sufficient variability in the ISSS to conduct individual differences analyses.

**Figure 7:**
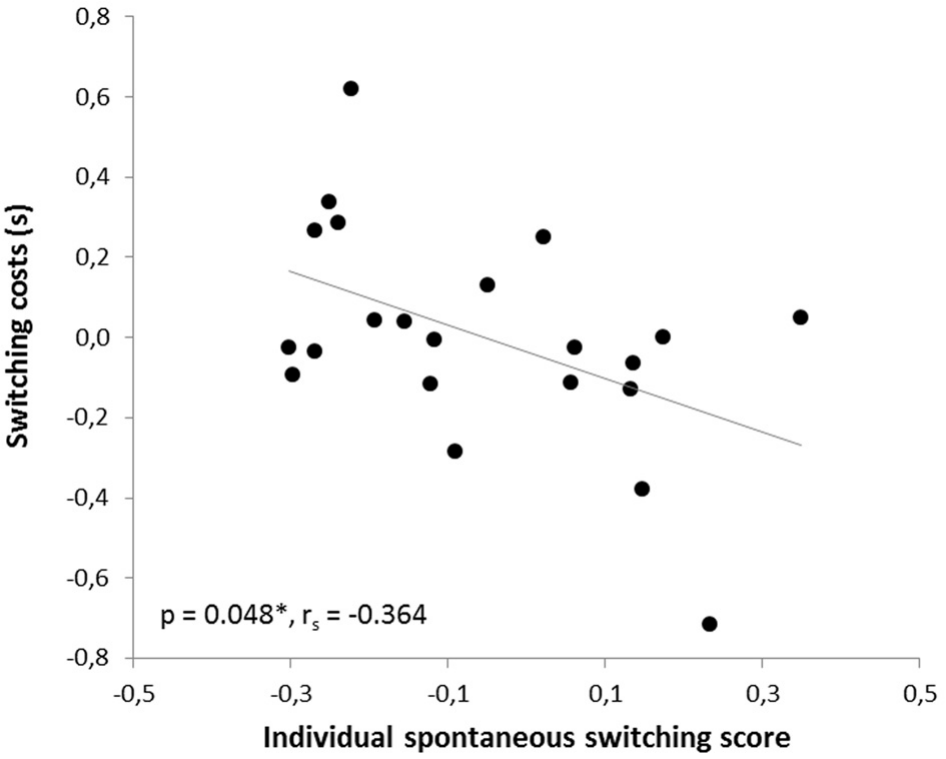
**Correlation between switching costs and the individual spontaneous switching rate**. Switching costs were calculated by subtracting response times of the ongoing condition from those of the switch condition. According to the predictions of the Dual State Theory, switching costs and the individual spontaneous switching score were negatively correlated: The more flexible the subject, the lower the switching costs in the switch condition. The correlation was calculated using Spearman's rank correlation coefficient.

**Figure 8:**
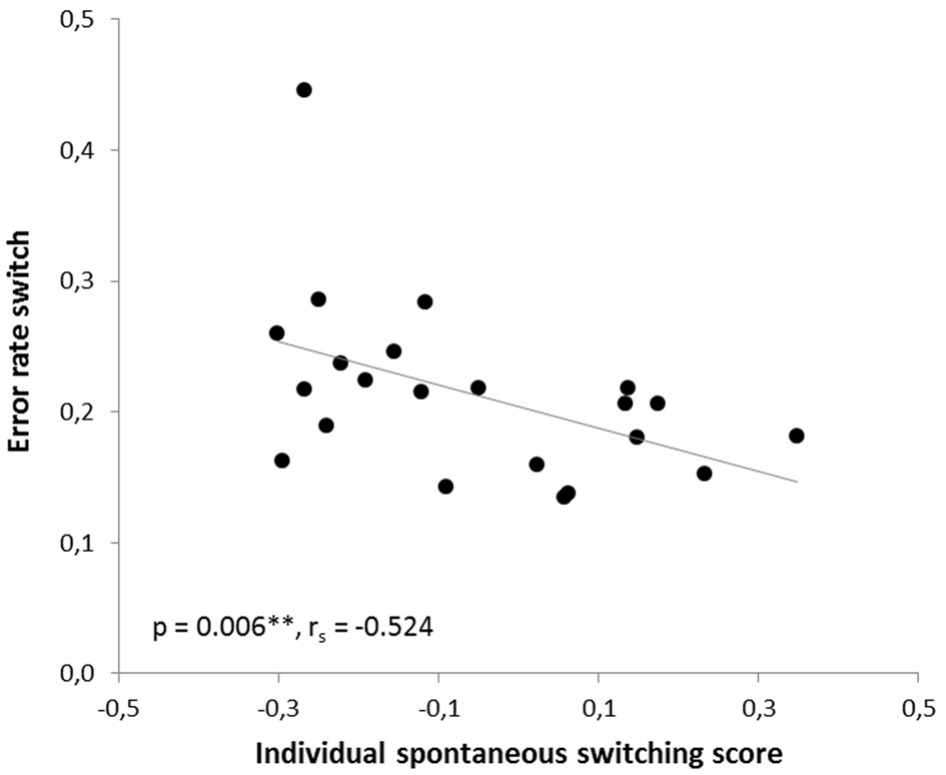
**Correlation between the error rate for switching and the individual spontaneous switching rate**. According to the predictions of the Dual State Theory, the error rate of the switch condition and the individual spontaneous switching score were negatively correlated: The more flexible the subject, the less errors it made in the switch condition. The correlation was calculated using Spearman's rank correlation coefficient.

**Figure 9:**
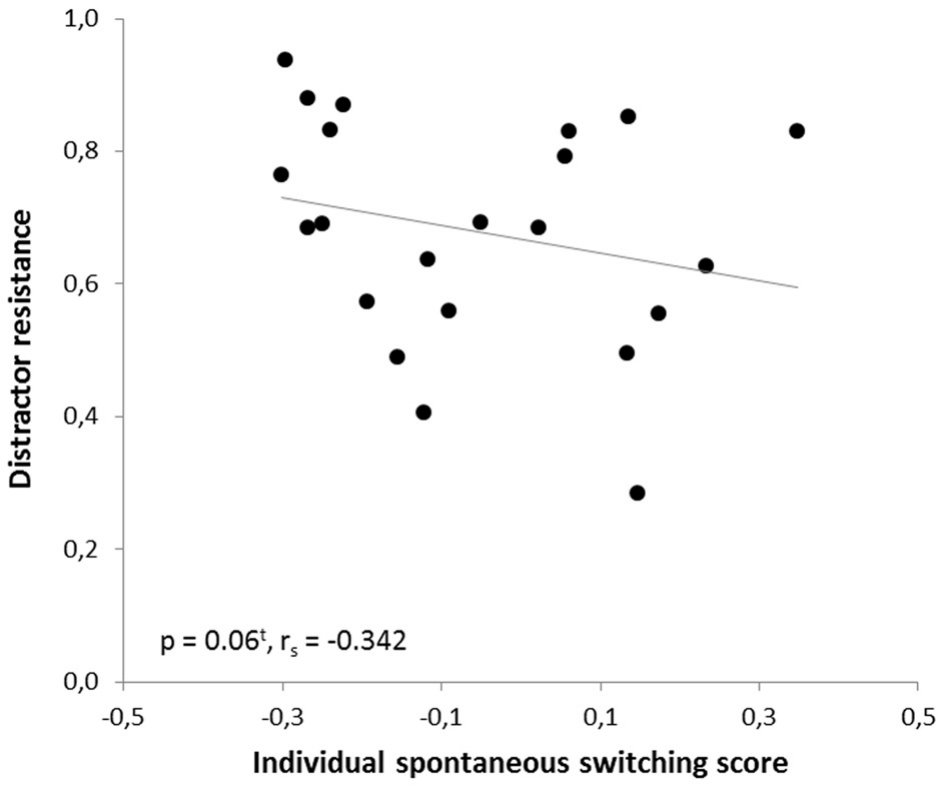
**Correlation between distractor resistance and the individual spontaneous switching rate**. According to the predictions of the Dual State Theory, the distractor resistance and the individual spontaneous switching score were negatively correlated: The more flexible the subjects, the less resistant to distractors they were. The correlation was calculated using Spearman's rank correlation coefficient.

In the human paradigm, the individual spontaneous switching rate was negatively correlated with the switching costs (Armbruster et al., 2012), assuming that this rate is not merely a reflection of a perceptual bias but that it indeed reflects a behavioral tendency toward more flexible behavior. Translating the test to the mouse condition led to a similar observation: Here, correlation analyses also revealed a significant negative correlation between the ISSS and the switching costs (*r* = −0.480, *p* = 0.012; *r*_*s*_ = −0.364, *p* = 0.048). Thus, the higher the ISSS and, thus, the more flexible the subject behaved in the “ambiguous” condition, the less “costly” it was for the subject to switch between the answers in non-ambiguous conditions when instructed to do so (Figure 7). According to further behavioral predictions, one should also expect a correlation between the ISSS and the error rate of the “switch” condition. Indeed, such a correlation was found with more flexible subjects making less switching errors under non-ambiguous conditions (Pearson product-moment correlation coefficient, *r* = −0.471, *p* = 0.0013; Spearman's rank correlation coefficient, *r*_*s*_ = −0.524, *p* = 0.006, Figure 8), even after the exclusion of one animal with an exclusively high error rate of 44.6% in the “switch” condition (*r* = −0.455, *p* = 0.019; *r*_*s*_ = −0.483, *p* = 0.013). However, no similar correlations were found between the ISSS and the ER of the “distractor switch” condition (*p* > 0.1). For distractor inhibition, the results were also in the expected direction, but less robust: Using Spearman's rank correlation coefficient, a trend toward a negative correlation between ISSS and distractor resistance was detected (*r*_*s*_ = −0.342, *p* = 0.06, Figure 9) that could, however, not be confirmed using Pearson's product-moment correlation coefficient (*r* = −0.237, *p* = 0.144).

## Discussion

In the present study, we introduce a novel task paradigm to assess cognitive flexibility vs. cognitive stability in mice. Inspired by neurocomputational modeling of the pFC-DA network, the task was originally established in a healthy human population (Armbruster et al., 2012), before being translated back to preclinical conditions in the present study.

Our aims were, first, to translate the procedure as analogously as possible from the human to the mouse, while at the same time accounting for species-specific demands, and, second, to test core behavioral predictions derived from the *Dual State Theory* (Durstewitz and Seamans, 2008) in a murine wildtype population, namely whether cognitive flexibility and stability are antagonistically related in individual subjects.

### Translating cognitive measures: the stabflex procedure

Translating tests of human executive functions still is a challenge (Tecott and Nestler, 2004). However, by providing virtually unlimited possibilities for task development, touchscreen approaches extend the repertoire for testing cognitive functions in animals like no other methodology (Bussey et al., 2012; Dickson et al., 2013; Horner et al., 2013; Mar et al., 2013; Oomen et al., 2013; Silverman et al., 2013; Talpos and Steckler, 2013). We carefully aimed to avoid confounding variables: Thus, the procedure is highly automated, avoids experimenter bias, and involves little stress for the animal (Chesler et al., 2002; Wahlsten et al., 2003; De Visser et al., 2006; Bussey et al., 2012; Strickland and Mercier, 2014). Moreover, all subjects were handled gently without physical restraint to reduce anxiety and stress (Hurst and West, 2010).

The cognitive task we established resembled the human version as closely as possible. As in the human paradigm, cues solely rely on visual input, which seems to be in contrast to rodents' low reliance on vision but can also be regarded as the strength of the system: By forcing the animals to solve problems in a “human-like” way, the approach has excellent face validity and prevents the experimental subjects from using secondary cues that we are unaware of (Talpos and Steckler, 2013).

Behavioral differences were assessed using response times and ER as it was done in the human study (Armbruster et al., 2012). By providing direct animal homologs of clinically important measures in human research, we aimed for high construct and predictive validity (Garner et al., 2006, 2011; Gould and Gottesman, 2006; Talpos and Steckler, 2013). Our test systematically investigated two cognitive domains, i.e., cognitive flexibility and cognitive stability, under the same conditions and using the same apparatus, thus completing the “wish-list” for an ideal cognitive testing method (Bussey et al., 2012).

However, there are also slight differences between the human and the animal paradigm, the most important being that while the switch condition of the human paradigm always involved the simultaneous presentation of a distracting cue, the mouse paradigm also involved trials with switching cues without the simultaneous presentation of a distracting cue. This was implemented to reduce complexity and adapt for the cognitive capacities of the mice (see below).

### The dichotomy of cognitive flexibility and cognitive stability

While behavioral differences between the task conditions could easily be detected on the basis of latency measures in the human paradigm, response time measurements were not sensitive enough to describe differences in the murine task version. Thus, average response times were two- to three-fold bigger in mice than in men, but varied less in mice. Probably, this discrepancy can be explained by the higher motoric component involved in the touching response. Humans were able to respond to the different task conditions by pressing a button within hundreds of milliseconds, while mice had to initiate the trial at the back of the chamber, then turning around, comparing the cues on the screen, before responding to the task by moving forward to either the left or the right cross. The high visual-motoric requirements may therefore overlap with the cognitive operations, challenging the use of response times in this context. This adds to previous discussions about the use of latency measures in cognitive testing (Richter et al., 2012).

ER, on the other hand, were very low and showed little variation in humans, but were well-suited to reflect the cognitive demands in mice. Although overall ER were much higher in mice than in men, similar differences between the conditions could be detected in both species. ER were found to increase from the “ongoing” to the “switch” and “distractor ongoing” conditions. This is in line with the findings of the human study reporting longer response times for task switching (= “distractor switch” in the murine study) than for distractor resistance = “distractor ongoing” in the murine study; Armbruster et al.(2012). Moreover, the data argue for the antagonistic model of cognitive flexibility and stability: Subjects with a higher ISSS were characterized by lower switching costs in the unambiguous “switch” condition. A similar correlation was found in the human task (Armbruster et al., 2012), validating the newly introduced measure of switching in an ambiguous condition (i.e., the ISSS) for mice. Furthermore, there was a significant correlation between the switching score and the error rate in the “switch” condition, indicating that more flexible subjects were able to switch more efficiently. In the “distractor switch” condition however, ER were highest and there was no correlation to the switching score, indicating that this task may be too complex for mice and thus is not suited to describing a mouse's disposition toward more flexible or stable behavior. We therefore believe that the mice's behavioral responses in the simple “switch” condition without distractor more adequately mimic what has been observed in the more complex human switch condition.

Finally, as more flexible mice also tended to be more distractible in the “distractor ongoing” condition, our results confirm the hypotheses that subjects switching more often spontaneously are more distractible to secondary cues, but also more flexible in situations, where cognitive flexibility is required. This is in line with previous findings on humans, showing that higher distractibility occurred in more flexible persons (Dreisbach and Goschke, 2004; Dreisbach et al., 2005; Tharp and Pickering, 2011; Armbruster et al., 2012; Zhang and Chan, 2013) and supports the predictions made by the *Dual State Theory* (Durstewitz and Seamans, 2008). Accordingly, the present study provides additional behavioral support from a different species for two crucial assumptions of the *Dual State Theory*, i.e., that individuals differ in their degree of cognitive flexibility and that cognitive flexibility and stability are antagonistically related—supposedly controlled by a common prefrontal neural mechanism involving differential effects of D1 and D2 receptor activation (Floresco and Phillips, 2001; Floresco and Magyar, 2006; Floresco et al., 2006; Vijayraghavan et al., 2007).

To validate the paradigm and to investigate the involvement of a pFC-DA network in the control of these higher executive functions, future studies are necessary, either working with dopamine-agonists/antagonists or with genetically modified mice to experimentally shift behavior toward a more stable or flexible pattern. In light of clinical research, this may contribute to a better understanding of cognitive dysfunctions in psychiatric diseases like schizophrenia and may even offer new routes for developing efficient pharmacological treatments in the long term.

## Conclusions

Here, we report the successful modification of a human neuropsychological task for use with mice. The results of this study support a dichotomy of cognitive flexibility and cognitive stability and thus confirm some fundamental predictions made by a biophysically realistic computational model of PFC function, i.e., the *Dual State Theory*. Similarities in behavioral patterns between humans and mice indicate the translational potential of the testing procedure and support the use of touchscreen procedures in preclinical animal research. This may launch an exciting new generation of behavioral tests for mice that promise to overcome many limitations of current high-throughput testing, while at the same time providing direct animal homologs of clinically promising measures in human research.

## Author contributions

S. Helene Richter, Christian J. Fiebach, Peter Gass, and Barbara Vollmayr conceived this work, S. Helene Richter, Anne S. Vogel, Miriam A. Vogt, Diana J. N. Armbruster-Genç, Marco A. Riva, Christian J. Fiebach, Peter Gass, and Barbara Vollmayr designed it, S. Helene Richter, Anne S. Vogel, Kai Ueltzhöffer, Chiara Muzzillo, and Katja Lankisch acquired data, S. Helene Richter, Anne S. Vogel, Kai Ueltzhöffer analyzed data under supervision and interpreted data together with Diana J. N. Armbruster-Genç, Christian J. Fiebach, Peter Gass, and Barbara Vollmayr. S. Helene Richter and Anne S. Vogel drafted the work, all authors critically revised it and approved the final version.

### Conflict of interest statement

The authors declare that the research was conducted in the absence of any commercial or financial relationships that could be construed as a potential conflict of interest.
